# Berberine Hampers Influenza A Replication through Inhibition of MAPK/ERK Pathway

**DOI:** 10.3390/v12030344

**Published:** 2020-03-21

**Authors:** Paweł Botwina, Katarzyna Owczarek, Zenon Rajfur, Marek Ochman, Maciej Urlik, Maria Nowakowska, Krzysztof Szczubiałka, Krzysztof Pyrc

**Affiliations:** 1 Virogenetics Laboratory of Virology, Malopolska Centre of Biotechnology, Jagiellonian University, 30-387 Krakow, Poland; pawel.botwina@doctoral.uj.edu.pl (P.B.); k.owczarek@uj.edu.pl (K.O.); 2 Department of Microbiology, Faculty of Biochemistry, Biophysics and Biotechnology, Jagiellonian University, 30-387 Krakow, Poland; 3 Institute of Physics, Faculty of Physics, Astronomy and Applied Computer Sciences, Jagiellonian University, Lojasiewicza 11, 30-348 Krakow, Poland; zenon.rajfur@uj.edu.pl; 4 Department of Cardiac Surgery and Transplantology, Silesian Center for Heart Diseases, Marii Curie-Skłodowskiej 9, 41-800 Zabrze, Poland; M.Ochman@sccs.pl (M.O.); m.urlik@sccs.pl (M.U.); 5 Faculty of Chemistry, Jagiellonian University, 30-387 Krakow, Poland; maria.nowakowska@uj.edu.pl (M.N.); k.szczubialka@uj.edu.pl (K.S.)

**Keywords:** influenza, berberine, MAPK pathway

## Abstract

Background: Berberine (BBR) is an isoquinoline alkaloid which exhibits a variety of biological and therapeutic properties, and has been reported by some to block replication of the influenza virus. However, contradictory results have also been presented, and the mechanistic explanation is lacking. Methods: A panel of cell lines (Madin–Darby canine kidney (MDCK), adenocarcinoma human alveolar basal epithelial cells (A549), lung epithelial type I (LET1)) and primary human airway epithelial cells (HAE) susceptible to influenza virus infection were infected with a seasonal influenza A virus in the presence or absence of BBR. Cytotoxicity towards cell lines was measured using XTT assay. The yield of the virus was analyzed using RT-qPCR. To study the molecular mechanism of BBR, confocal microscopy and Western blot analyses of cellular fractions were applied. Results and conclusions: Our results show cell-type-dependent anti-influenza properties of BBR in vitro which suggests that the compound acts on the cell and not the virus. Importantly, BBR hampers influenza replication in primary human airway epithelium 3D cultures that mimic the natural replication site of the virus. Studies show that the influenza A virus upregulates the mitogen-activated protein kinase/extracellular signal-related kinase (MAPK/ERK) pathway and hijacks this pathway for nucleolar export of the viral ribonucleoprotein. Our results suggest that BBR interferes with this process and hampers influenza A replication.

## 1. Introduction

The influenza viruses are among the most important human pathogens, with types A and B being the most clinically relevant for humans [1,2], and type A being the sole cause of pandemics. Influenza is a severe, acute, and highly contagious respiratory system disease. The symptoms include high fever, chills, general weakness, diffuse headaches, runny nose, muscle aches, sore throat, dry paroxysmal cough, and conjunctivitis. It is estimated that yearly seasonal influenza causes 3 to 5 million severe cases, and these are associated with about 300,000 to 650,000 deaths [3].

Influenza A viruses infect a wide variety of species [4], and currently, two strains are believed to circulate in humans (A(H1N1) and A(H3N2)), while a number of strains reside in birds and mammals. Of these, sporadic transmissions to humans occur and are frequently characterized by high pathogenicity [5]. The key factor that makes the influenza virus unique is its variability and the genome present in virions in eight segments. Co-infection of a single cell results in shuffling of these genomic RNA puzzles, and new, hybrid progeny emerges. Reassortants are usually less fit and are eliminated, but in sporadic cases, a new strain appears that is able to effectively infect human cells but is not recognized by our defense systems.

WHO recommends vaccination as the most effective way to prevent the disease, as this provides ~70%–90% of protection [6,7]. However, the vaccine does not provide complete protection, especially in infants, the elderly, and patients with immune deficits. Antivirals are, therefore, a complementary tool for the comprehensive management of the disease—five approved anti-influenza drugs are available. The old generation of drugs, amantadine and rimantadine, interfere with M2 protein activity and inhibit the release of the viral genome to the replication site during virus entry. Unfortunately, due to the emergence of drug-resistant variants, these medicinal products have already been withdrawn [8]. The second group of drugs encompasses zanamivir and oseltamivir, which inhibit the activity of the neuraminidase (NA) protein. These NA inhibitors (NAIs) interfere with the virus egress and, to a lesser extent, also entry. However, the emergence of drug resistance has been already reported [8]. In October of 2018 the FDA approved the new anti-influenza active substance, Baloxavir marboxil (Xofluza). Baloxavir inhibits viral endonuclease, which is responsible for cleavage of cellular mRNAs near their 5′-termini, in order to generate the primers required for viral RNA synthesis (cap snatching) [9]. Nonetheless, as clearly shown for, e.g., the HIV-1 virus, only a combined therapy, simultaneously interfering with different molecular targets, is effective and long-lasting.

Berberine (BBR) is an isoquinoline alkaloid that belongs to the structural class of protoberberines and is produced by plants, including *Berberis vulgaris* and *Coptis sp.* [10]. BBR has a long history as a traditional remedy in many parts of the world [11]. The activity of BBR has been widely discussed in the scientific literature—it has been reported to exhibit therapeutic properties including anti-inflammatory, antioxidant, anti-cancer, anticonvulsant, antidepressant, anti-Alzheimer, anti-arrhythmic, and anti-diabetic effects in vitro and in vivo [12,13]. Moreover, the compound exhibits broad-spectrum antimicrobial activity against bacteria and viruses. BBR has been reported to be effective against *Streptococcus agalactiae*, *Actinobacillus pleuropneumoniae*, and *Staphylococcus spp*. Additionally, BBR has been shown to block herpes simplex virus types 1 and 2 (HSV-1, 2) replication in Vero cells [14]. Recent studies have shown potent inhibition of the Zika virus (ZIKV) [15,16]. Other reports have shown BBR-mediated suppression of respiratory syncytial virus (RSV) replication in epithelial cells, probably via inhibition of RSV-mediated early p38 mitogen-activated protein kinase (MAPK) activation and inhibition of virus entry into the cell [17]. Another publication showed an inhibitory effect on the Chikungunya virus (CHIKV) through inhibition of virus-induced MAPK signaling [10,13,18].

The literature claims that BBR also inhibits the replication of the influenza virus. In the work by Cecil et al., BBR inhibited the replication of two strains of the H1N1 virus in a murine macrophage cell line (RAW 264.7) and adenocarcinoma human alveolar basal epithelial cells (A549) but did not prevent the expression of viral proteins [19]. The authors reported that virus replication was diminished by inducing the formation of viral protein aggregates within the host cell cytoplasm. An in vivo experiment (mice models) confirmed these observations, as BBR reduced mice mortality from 90% to 55% and decreased virus titers in the lungs on day 2 post-infection (p.i.) [20,21]. Moreover, BBR also suppresses pulmonary inflammation and reduces necrosis, inflammatory cell infiltration, and pulmonary edema [21]. Cecil et al. tested the anti-influenza properties of BBR using three in vitro models, i.e., A549 cells, RAW 264.7 cells, and Madin–Darby canine kidney (MDCK) cells and observed inhibition of virus replication only in the first two cell types [19]. No inhibition was noted in MDCK cells. On the other hand, Wu et al. tested BBR only in MDCK cells and observed strong inhibition of virus replication [20]. 

Despite numerous reports on BBR activity, the mechanism of its antiviral action is far from being understood. Our results show strong anti-influenza properties of BBR in vitro and ex vivo, and we believe that this observed effect is relevant in the human body. Moreover, we show that BBR blocks the influenza-induced mitogen-activated protein kinase/extracellular signal-related kinase (MAPK/ERK) pathway, which is required for the transport of viral ribonucleoproteins to the cytoplasm.

## 2. Materials and Methods 

### 2.1. Compounds

Berberine chloride hydrate (BBR) was obtained from Sigma–Aldrich, Poznan, Poland. Stock solutions of BBR (50 mM) were prepared by dissolving BBR in dimethyl sulfoxide (DMSO) (Sigma–Aldrich, Poznan, Poland.) The fresh stock was prepared before each experiment. U0126 stock (Invivogen, Toulouse, France), a specific inhibitor of the extracellular signal-related kinase (ERK), was dissolved in DMSO (25 mM), aliquoted and stored at −20 °C. 

### 2.2. Virus and Cells

Madin–Darby canine kidney (MDCK) cells (ATCC CCL-34 cell line), adenocarcinoma human alveolar basal epithelial cells (A549) (ATCC CCL-185 cell line), and lung epithelial type I (mouse) immortalized cell line (LET1) (NR-42941, BEI Resources, Manassas, VA, USA) were maintained in Dulbecco’s modified Eagle’s medium (DMEM, high glucose, Life Technologies, Eugene, OR, USA) supplemented with 3% heat-inactivated fetal bovine serum (FBS, Life Technologies, USA), penicillin (100 U/mL), and streptomycin (100 μg/mL) (3% DMEM). Cells were cultured at 37 °C in an atmosphere containing 5% CO_2_. 

Human airway (tracheobronchial) epithelial (HAE) cells were obtained from airway specimens resected from patients undergoing surgery at the Silesian Center for Heart Diseases. The study was approved by the Bioethical Committee of the Medical University of Silesia in Katowice, Poland (approval no: KNW/0022/KB1/17/10 dated 16 February 2010). Written consent was obtained from all patients. Primary cells were cultured on plastic to generate passage 1 cells and plated at a density of 3 × 10^5^ cells/well on permeable Transwell inserts supports. HAE cultures were generated by the provision of an air–liquid interface for 6 to 8 weeks to form fully differentiated, polarized cultures that resemble pseudostratified mucociliary epithelium.

Human seasonal influenza virus type A H3N2, strain A/Hong Kong/4801/2014, was obtained from the European Virus Archive EVA project (EVAg, Marseille, France). Virus stocks were generated by infecting MDCK cells at 90% confluency for 48 h. After that time, cultures were aliquoted and stored at −80 °C. Mock samples were prepared in the same manner, using uninfected cells. Virus stocks were quantified by titration, as previously described [22].

### 2.3. Replication Inhibition Assay

MDCK, A549, or LET1 cells were seeded in 96-well plates (TPP, Trasadingen, Switzerland) at the density of 10^4^ cells per well. After 24 h, cells were infected with influenza A virus at a dose of 400 50% tissue culture infectious dose (TCID_50_)/mL in the presence of BBR in a total volume of 100 μL in DMEM supplemented with penicillin/streptomycin and TPCK-treated trypsin 1 µg/mL (Sigma–Aldrich, Poznan, Poland). In the case of the HAE cultures, virus and BBR were applied to the apical surfaces of the HAE cultures. Control samples were inoculated in the same manner with the same volume of mock and/or DMSO. After 2 h of incubation at 37 °C, cells were rinsed thrice with PBS and fresh medium supplemented with BBR was added. The infection was carried out for 48 h (cell lines) or 72 h (HAE). Virus yield was measured using the RT-qPCR method described below.

### 2.4. Mechanism of Action Assays

To determine whether BBR affects a particular stage of the replication cycle of the virus, a series of previously described mechanistic experiments [22,23] was carried out. All assays were conducted on sub-confluent LET1 cells.

(I) Virus inactivation assay shows the influence of tested compounds on the viral particle. Influenza A virus stock was incubated with BBR under constant mixing for 1 h at room temperature. Samples were diluted 100 times to ensure that the BBR concentration was below the lower limit of the effective range. Samples were then titrated according to the Reed and Muench method [24].
(II) Cell protection assay examines the effect of the compound on the cell surface. In this assay, cells were incubated with BBR for 2 h at 37 °C. After incubation, the media were removed, and cells were washed with PBS. Then, cells were infected with the influenza A virus (TCID_50_ = 400) or mock control for 2 h at 37 °C. Next, media were discarded, cells were washed thrice with PBS, and fresh infection medium was added. Cells were incubated for 48 h at 37 °C.
(III) Virus attachment assay examines the effect of the compound on the virus–receptor interaction. Confluent cells were pre-cooled to 4 °C and overlaid with ice-cold influenza A virus in BBR-containing medium (TCID_50_ = 400) or mock control. Samples were incubated at 4 °C for 1 h to allow for virus attachment to the host cell but not for virus internalization [25]. Then, cells were rinsed thrice with cold PBS to remove the residual virus and fresh medium was applied. Cells were incubated for 48 h at 37 °C.
(IV) Virus internalization assay for evaluation of virus entry into the susceptible cell. Pre-cooled, confluent cells were infected with a virus (TCID_50_ = 400; ice-cold solution) for 2 h at 4 °C to avoid virus internalization to cells. After incubation, the medium was discarded, and cells were washed thrice with cold PBS. Then, media supplemented with BBR were applied, and samples were incubated for 2 h at 37 °C to allow for virus internalization. Next, cells were washed with an acidic buffer (pH 3.0; 0.1 M glycine, 0.1 M sodium chloride) to inactivate uninternalized virions. Cultures were rinsed once with PBS and fresh media were applied. Cells were incubated for 48 h at 37 °C.
(V) Virus replication, assembly, and egress assay evaluates virus replication and production of infectious progeny. Cell cultures were infected with influenza A virus (TCID_50_ = 400) for 2 h at 37 °C. Then, the residual virus was washed out thrice with PBS. Medium supplemented with BBR was applied, and cells were incubated for 48 h at 37 °C.

### 2.5. Cell Viability 

Cell viability was evaluated using the XTT Cell Viability Assay kit (Biological Industries, Cromwell, CT, USA) according to the manufacturer’s instructions. Cells were incubated with BBR for 48 h at 37 °C in an atmosphere containing 5% CO_2_. After incubation, the medium was discarded and 100 μL of fresh medium was added to each well. Then, 50 μL of the activated 2,3-bis-(2-methoxy-4-nitro-5-sulphenyl)-(2H)-tetrazolium-5-carboxanilide (XTT) solution was added and samples were incubated for 2 h at 37 °C. The absorbance (λ = 450 nm) was measured using a Spectra MAX 250 spectrophotometer (Molecular Devices, San Jose, CA, USA). Data were presented as the ratio of signal from the examined sample and from the control sample (solvent-treated cells) × 100%.

### 2.6. Confocal Microscopy and Image Analysis

For confocal microscopy analysis, 0.1 × 10^6^ A549 cells were seeded on coverslips in 12-well plates (TPP, Trasadingen, Switzerland). After 12 h, cells were washed with PBS and infected with influenza A virus in the presence of BBR. 

After incubation at 37 °C, cells were washed with PBS, fixed with 4% paraformaldehyde, permeabilized with 0.1% Triton X-100, and incubated for 2 h with 5% bovine serum albumin (BSA) containing 0.1% Tween 20 (Sigma–Aldrich, Poland). Subsequently, cells were incubated for 2 h with mouse anti-nucleoprotein antibody (1 μg/mL; BEI Resources, Manassas, VA, USA) diluted 1:250 in Tris-buffered saline (TBS)-Tween (0.1%) buffer. Then, cells were incubated for 1 h with Alexa Fluor 488-labeled goat anti-mouse antibody (2.5 μg/mL; Molecular Probes, Eugene, OR, USA) diluted 1:1000 in Tris-buffered saline (TBS)-Tween (0.1%) buffer. Nuclear DNA was stained with DAPI (4’,6-diamidino-2-phenylindole; 0.1 μg/mL; Sigma–Aldrich, Poznan, Poland). Fluorescent images were acquired using a Zeiss LSM 880 confocal microscope (Carl Zeiss Microscopy GmbH, Jena, Germany) and analyzed with ImageJ Fiji software [26]. The relative amount of influenza A nucleoprotein present in the nuclei was assessed by scoring the fluorescence signal of the viral protein overlapping with the nuclei. The data were presented as the proportion of fluorescence intensity in the nuclei to the total cell fluorescence intensity. 

Distribution of values was tested using the Shapiro–Wilk normality test, and equality of group variances was examined with Browne–Forsythe test. Statistical significance was calculated using Kruskal–Wallis test with Dunn’s multiple comparisons test. *p* values <0.05 were considered significant and denoted with an asterisk. 

### 2.7. Quantitative Real-Time PCR 

Viral RNA was isolated using the Viral DNA/RNA Isolation Kit (A&A Biotechnology, Gdynia, Poland) according to the manufacturer’s instructions. Then, reverse transcription was carried out with a high-capacity cDNA reverse transcription kit (Applied Biosystems, Waltham, MA, USA) according to the manufacturer’s instructions using a Veriti thermal cycler (Applied Biosystems, Waltham, MA, USA). The whole cDNA was determined by quantitative real-time PCR (qPCR). The reaction was carried out in a CFX96 Touch Real-Time PCR Detection System (Bio-Rad, Hercules, CA, USA) (2 min at 50 °C, 10 min at 95 °C, followed by 40 cycles of 15 s at 95 °C and 1 min at 60 °C). The reaction mixture contained 1 × RT HS-PCR mix probe (A&A Biotechnology, Gdynia, Poland), a specific probe labeled with 6-carboxyfluorescein (FAM), a black hole quencher 1 (BHQ1) (sequence 5′- TCA GGC CCC CTC AAA GCC GA-BHQ1-3′, 100 nM), primers specifically targeting the influenza A matrix protein (450 nM each, sense primer 5′-AGA TGA GTC TTC TAA CCG AGG TCG-3′, antisense primer 5′-TGC AAA AAC ATC TTC AAG TCT CTG-3′), and 2.5 μL of viral cDNA in 10 μL reaction mixture. For virus copy number quantification a standard DNA template of the known copy number was prepared and serially diluted, and the standard curve was prepared as described previously [22]. The lower limit of detection was < 10^3^ copies/mL. 

### 2.8. Isolation of Nuclear and Cytoplasmic Proteins

Nuclear and cytoplasmic fractions were isolated as described before [27]. Briefly, A549 cells were washed once with PBS and resuspended in hypotonic buffer A: 20 mM Tris HCl [pH 7.5], 10 mM NaCl, 3 mM MgCl_2_, 10% glycerol, and a protease inhibitors cocktail (Roche, Warsaw, Poland) for 1 min. Then, NP-40 was added to reach 0.1% *v/v* concentration and samples were incubated for 5 min. After centrifugation (600× *g* at 4 °C), the cytoplasmic fraction was collected. Then, nuclei were resuspended in nuclear extraction buffer B: 20 mM Tris-HCl pH 7.5, 400 mM NaCl, 3 mM MgCl_2_, 20% glycerol. After 30 min on ice, nuclei were subjected to three cycles of snap-freeze/thaw, and insoluble proteins were removed from the nuclear extract by high-speed centrifugation at 4 °C. Obtained fractions were mixed with sample buffer (0.5 M Tris, pH 6.8, 10% SDS, 50 mg/mL dithiothreitol (DTT)), boiled for 5 min, and separated on 10% polyacrylamide gels alongside dual-color PageRuler prestained protein size markers (Thermo Scientific, Warsaw, Poland). The separated proteins were then transferred onto a Westran S polyvinylidene difluoride (PVDF) membrane (Whatman Maidstone, UK) by wet blotting (Bio-Rad, Hercules, CA, USA) for 1.5 h at 100 V in transfer buffer containing 25 mM Tris, 192 mM glycine, and 20% methanol at 4 °C. The membranes were then blocked by overnight incubation (at 4 °C) in Tris-buffered saline (TBS)-Tween (0.1%) buffer (TTBS) supplemented with 5% skimmed milk (BioShop, Burlington, ON, Canada). Membranes were incubated for 2 h with mouse anti-NP primary antibodies (BEI Resources, Manassas, VA, USA) diluted 1:250. For evaluation of fractions purity, rabbit anti-histone 3 and GAPDH-diluted 1:1000 antibodies (Thermo Scientific, Warsaw, Poland) were used. Then, suitable secondary antibodies labeled with horseradish peroxidase (Santa Cruz Biotechnology, Dallas, TX, USA) were used (1:10,000). All antibodies were diluted in TBS-Tween (0.1%) supplemented with 1.5% skimmed milk. The signal was developed using an Immobilon Western Chemiluminescent HRP (Horseradish Peroxidase) Substrate (Merck Millipore, Warsaw, Poland) and visualized by ChemiDoc™ MP Imaging System (Bio-Rad, Hercules, CA, USA).

### 2.9. Statistics

The results are expressed as means ± standard deviations (SD). The half-maximal inhibitory concentration (IC_50_) and 50% toxic concentration (TC_50_) values were calculated using the Graph Pad Prism 8.0 function (GraphPad Software, San Diego, CA, USA). The selectivity index (SI) was defined as the TC_50_/IC_50_ ratio. To determine the significance of the results obtained, Student’s t-test was used and *p* values < 0.05 were considered significant. Western blot analyses were conducted using Image Lab 6.0 (Bio-Rad, Hercules, CA, USA). 

## 3. Results 

### 3.1. The Antiviral Effect of BBR Is Cell-Type Dependent

**Figure 1:**
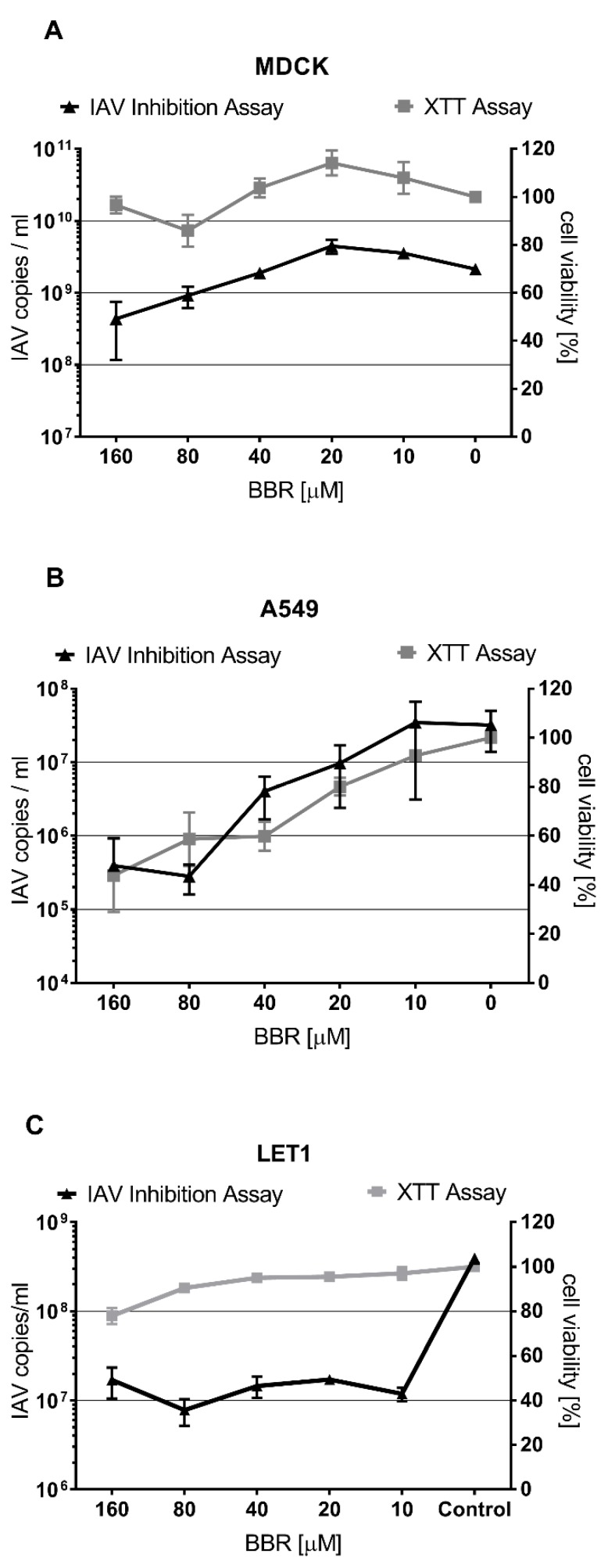
BBR-mediated inhibition of influenza A virus is cell-type dependent. RT-qPCR analysis (black, left Y-axis) of cell culture supernatants infected with influenza A virus (IAV); tissue culture infectious dose (TCID_50_) = 400/mL) with or without BBR. Control samples were treated in the same manner with the same volume of DMSO. XTT assay results (right Y-axis) are indicated in gray (**A**–**C**). The results are presented as average values with standard deviations (error bars). All experiments were performed at least in triplicate.

To investigate the antiviral activity of BBR in vitro, a series of cell lines permissive to the influenza A virus were infected in the presence of BBR (10–160 µM). First, the most common in vitro model was used, i.e., the cell line derived from Madin–Darby canine kidney cells (MDCK). We performed a standard virus replication assay by infecting the MDCK cells in the presence or absence of BBR. Concomitantly, cell viability was monitored using the XTT assay. A significant inhibitory effect of BBR in the MDCK cells was observed in our experimental setting in samples treated with BBR at 80 and 160 µM, while IC_50_ was equal to 52 µM. We did not observe any significant decrease in cell viability (TC_50_ = 1035 µM, SI = 20) (Figure 1A, Table 1). These results indicate inhibitory properties similar to those obtained by Wu et al. (IC_50_ = 0.025 µg/mL ≈ 67 µM). We conclude that BBR hampers replication of the influenza A virus in MDCK cells, but to a much lesser extent than in other in vitro models [19,28]. 

Next, the human pulmonary tumor cell line A549 was assayed, as inhibition of the influenza virus by BBR in this cell line has been consistently reported [19,21]. Many reports show a high toxicity of BBR to cancer cells, including the A549 cell line [29,30], therefore BBR has been proposed as an antineoplastic drug candidate. Considering that BBR cytotoxicity was not assayed in these cells, we decided to verify the antiviral BBR activity in A549 cells. Our results indicate a significant decrease in influenza A replication in the presence of BBR, but the compound was cytotoxic at the active concentrations (IC_50_ = 17 µM, TC_50_ = 107 µM, SI = 6) (Figure 1B, Table 1). 

Finally, we decided to test the immortalized murine lung epithelial type I cell line (LET1). This cell line is non-cancerous and maintains most of the natural signaling pathways [31]. First, we verified the cytotoxicity of BBR on these cells and it was acceptable (TC_50_ = 521 µM). Next, we tested the effect of BBR on the replication of the influenza A virus in LET1 cells. RT-qPCR analysis revealed a vast decrease in viral replication at non-toxic concentrations (IC_50_ = 4 µM, SI = 123) (Figure 1C, Table 1). 

**Figure 2:**
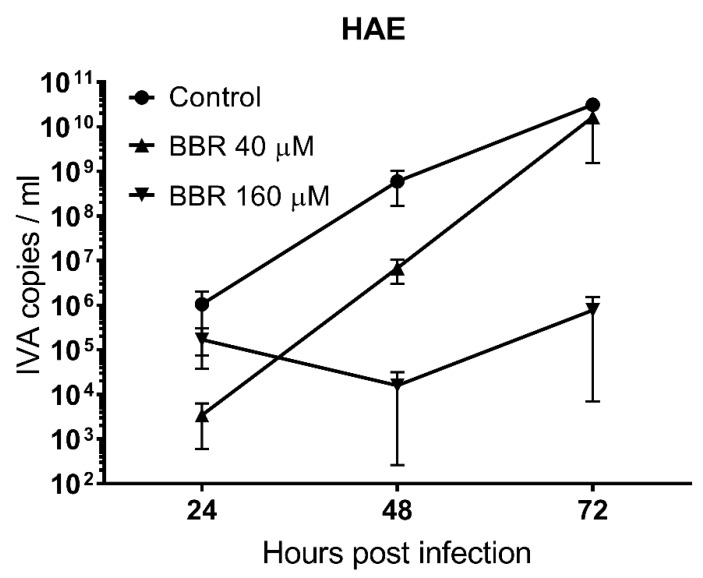
BBR-mediated inhibition of influenza A virus in fully differentiated HAE cultures. RT-qPCR analysis (black, left Y-axis) of cell culture or tissue culture supernatants infected with influenza A virus (IAV); TCID_50_ = 400/mL) with or without BBR at various time points. Control samples were treated in the same manner with the same volume of DMSO. The results are presented as average values with standard deviations (error bars). All experiments were performed at least in triplicate.

To summarize, we have tested three different in vitro models, and we have obtained three different results. To sort out the actual activity of BBR, we decided to evaluate BBR in a more natural system, mimicking the human airway epithelium (HAE). Briefly, HAE was reconstituted on the interface of air and media using primary human cells. This model has previously been well-characterized and successfully used by us in other studies [22,32,33]. To the best of our knowledge, it is the most natural cellular model for testing of potential inhibitory compounds against a variety of respiratory viruses. 3D HAE cultures form well-differentiated, polarized cultures that resemble in vivo pseudostratified mucociliary epithelium lining the conductive airways of the human respiratory tract. We did not observe cytotoxicity at tested concentrations (i.e., up to 160 µM, 0.054 µg/mL), suggesting that BBR is not toxic towards non-cancerous cells, consistent with previously published data. However, BBR inhibited influenza A virus replication in HAE cultures only at higher concentrations compared to LET1 cells (IC_50_ = 16 µM). This may be explained by the fact that BBR was not continuously present on the cultures, but only during daily washes of the apical surface (Figure 2).

Taken together, BBR is expected to inhibit the influenza A virus during the natural infection, but care should be taken during the selection of the in vitro model.

### 3.2. BBR Inhibits the Influenza A Virus Replication at Late Stages of the Infection 

**Figure 3:**
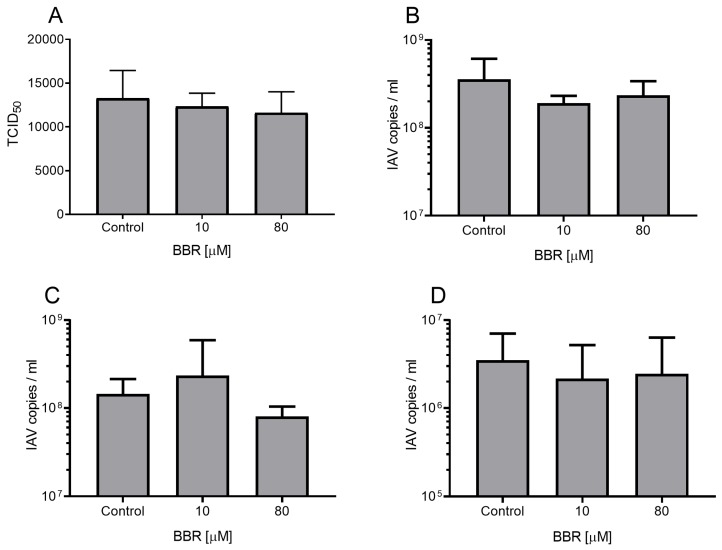
BBR does not inhibit the early steps of influenza A virus infection. Modulation of virus yield as determined by titration (**A**) or RT-qPCR (**B**) after the virus inactivation assay (I), where the virus was pre-incubated with BBR or DMSO, diluted and titrated on MDCK cells. Modulation of virus yield as determined by RT-qPCR after the receptor attachment assay (**C**) or virus internalization assay (**D**). In assays B–D, LET1 cells were infected with influenza A virus (IAV); TCID_50_ = 400/mL) and BBR was added at different stages of the virus replication cycle. Control samples were treated in the same manner with the same volume of DMSO. The results are presented as average values of three replicates with standard deviations (error bars).

**Figure 4:**
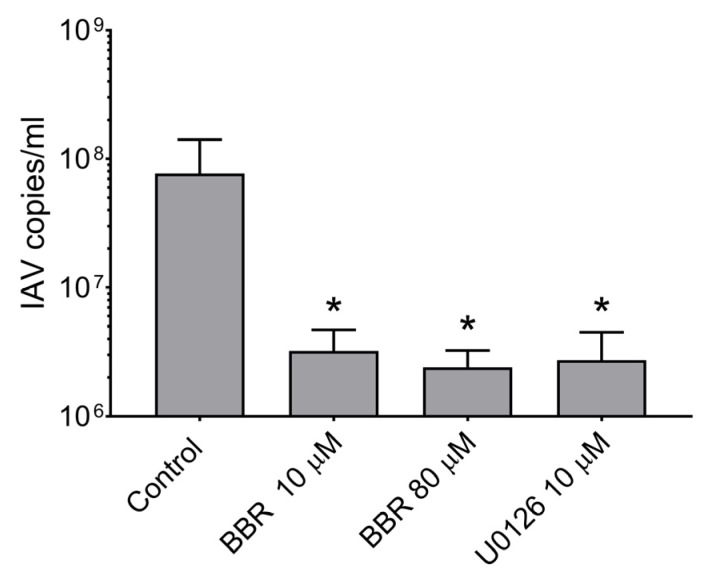
BBR inhibits influenza A replication at the late stages of infection. LET1 cells were infected with influenza A virus (IAV); TCID_50_ = 1000/mL). After 2 h p.i., 10 µM U0126 in DMSO or BBR were added. Control samples were treated in the same manner with the same volume of DMSO. All experiments were performed in triplicate. The results are presented as average values with standard deviations (error bars). An asterisk (*p* < 0.05) indicates values that are significantly different from the control.

As we confirmed the anti-influenza activity of BBR, our next step was to determine whether BBR affects a particular stage of the virus replication cycle. To investigate this, we made a series of mechanistic experiments as described in our earlier works [22,23]. Our assays I–IV did not show a significant decrease in the influenza A virus RNA copy number measured with RT-qPCR, suggesting that BBR did not interfere with the virus itself, virus entry, trafficking, and genome replication (Figure 3). However, we observed a decrease in assay V, which suggests that BBR affects virus assembly, maturation, or egress (Figure 4). 

### 3.3. BBR Inhibits the Influenza A Virus Replication through Downregulation of the MAPK/ERK Pathway

Data presented above and recently published works on other viruses suggest that BBR acts on cellular pathways that are essential for efficient virus replication. 

**Figure 5:**
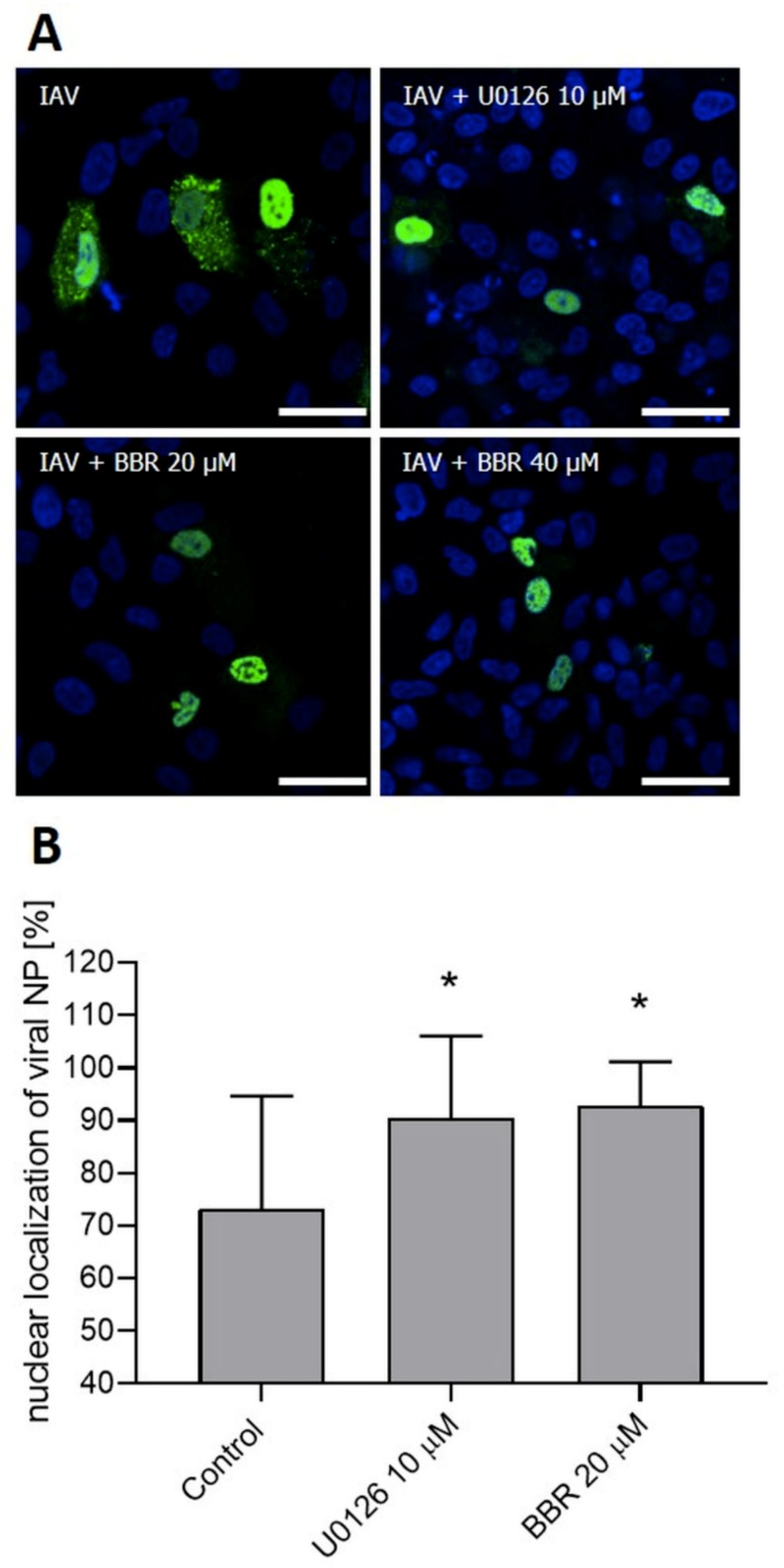
BBR blocks the transport of influenza A ribonucleoprotein to the cytoplasm. A549 cells were infected with influenza A virus (IAV); TCID_50_ = 2000/mL) in the presence of 10 µM U0126 in DMSO (U0126), 20 µM BBR (BBR 20 µM), or 40 µM BBR (BBR 40 µM). Cells were fixed 12 h after the infection, and confocal images were collected (**A**). Cell nuclei are denoted in blue, influenza A NP protein is denoted in green. Scale bar = 50 µm. Confocal microscopy analysis of the nuclear localization of influenza A virus nucleoprotein (**B**). The statistical significance of the phenomena was assessed by image analysis, where retention of influenza A virus nucleoprotein in nuclei was scored as described in the Materials and Methods section. The data are presented as mean value ± SD from at least four hundred cells collected from at least three independent experiments. An asterisk (*p* < 0.05) indicates values that are significantly different from the control.

**Figure 6:**
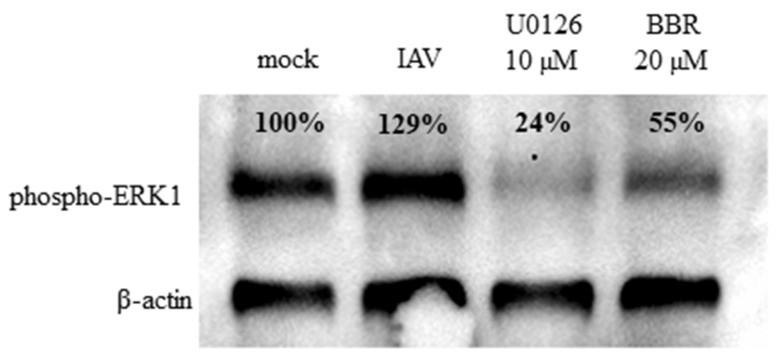
BBR inhibits virus-induced mitogen-activated protein kinase/extracellular signal-related kinase (MAPK/ERK) pathway. A549 cells were infected with influenza A virus (IAV); TCID_50_ = 2000/mL) or with mock with the addition of DMSO solvent, U0126 (10 µM), or BBR (20 µM). Cells were lysed 12 h after infection and resolved by 12% SDS-PAGE. Phosphorylated ERK-1 protein was visualized by Western blot with an anti-phosphorylated ERK-1 antibody. *β*-actin was visualized as a reference control. Percentage values show the relative expression ratio of phospho-ERK1 after normalization to *β*-actin signal in each lane.

**Figure 7:**
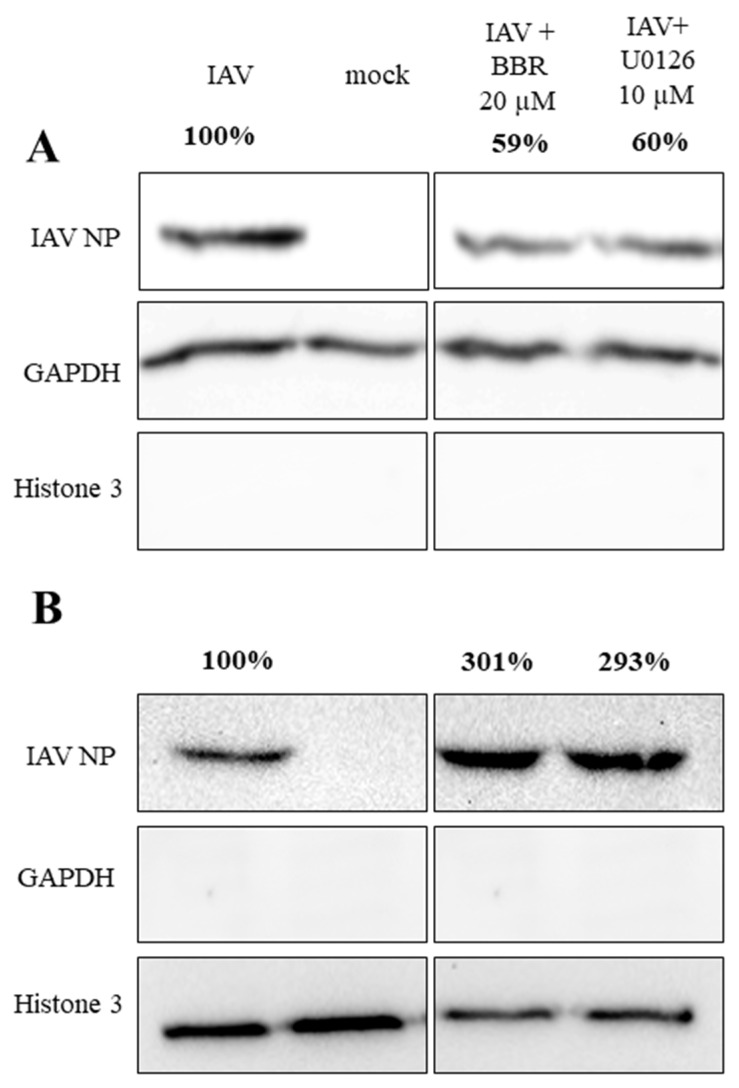
BBR treatment leads to the accumulation of influenza A virus NP in nuclei. The figure shows Western blot analyses of cytoplasmic (**A**) and nuclear (**B**) fractions of A549 cells infected with influenza A virus (TCID_50_ = 2000/mL) with the addition of DMSO solvent, U0126, or BBR at given concentrations. After 12 h, cytoplasmic and nuclear fractions of the cells were obtained and resolved by 12% SDS-PAGE. Influenza A nucleoprotein (IAV NP) was visualized by Western blot with anti-NP. GAPDH (glyceraldehyde 3-phosphate dehydrogenase) and histone 3 proteins were blotted as fraction purity markers. Percentage values show relative expression ratio of influenza A virus NP after normalization to GAPDH (A) or histone 3 (B) signal in each lane.

To confirm whether BBR inhibits the influenza A virus through downregulation of the MAPK/ERK pathway, confocal imaging of infected A549 cells treated with two different concentrations of BBR and with an active concentration of specific MAPK/ERK inhibitor U0126 (10 µM) was conducted. After 12 h, we observed characteristic accumulation of a viral RNP complex inside nuclei, in both cells treated with BBR and U0126 (Figure 5A). This effect was confirmed by image analysis. Significant differences in NP signal accumulation were observed for both the U0126 and BBR cells compared to the untreated control (Figure 5B). More than 90% of NP colocalized with nuclear staining in cells treated with U0126 or BBR compared to about 70% in control cells. This effect was clearly visible between 12 and 20 h p.i. After this time, large amounts of RNP were also visible in the cytoplasm (data not shown). This outcome may be due to the removal of BBR from the cell or its degradation. Western blot analyses indicate moderate inhibition of ERK1 activation by BBR at 20 µM concentration (45% inhibition) compared to the U0126 cell (76% inhibition) (Figure 6.). We observed, however, a slight increase in the amount of phospho-ERK in control-infected cells, as previously reported [34]. To confirm confocal imaging results, we performed a similar experiment and isolated cytoplasmic and nuclear fractions for Western blot analysis. The analysis showed higher quantities (~3 fold increase) of viral NP in nuclear fractions of cells treated with U0126 or 20 µM BBR (0.007 µg/mL) compared to untreated infection, confirming our confocal imaging observations (Figure 7B). Lower BBR concentration, however, did not cause significant accumulation of influenza A virus NP (data not shown). In the case of cytoplasmatic fraction, we observed a moderate yet clear decrease of NP (about 40% inhibition) signal in treated cells which may suggest that BBR does not interfere with the assembly and release of virions. Taking these results together, obtained data suggest an inhibition on the influenza A virus by BBR through the downregulation of the MAPK/ERK1 pathway. 

## 4. Discussion

Influenza A virus infections are among major public health threats. Despite many efforts, the list of approved drugs is short, and the emergence of resistance is rapid [8]. Consequently, there is a continual need for new, broad-spectrum, anti-influenza drugs, and many different sources for such drugs are considered. Natural products constitute a rich library of compounds, which may be used to identify new structures and new molecular targets. However, knowledge of the mechanism of action is essential. Berberine (BBR) exhibits a number of biological activities and, among others, has been described as a potent inhibitor of the influenza virus [12]. In this work, we undertook a comprehensive study of the antiviral properties of BBR in vitro and ex vivo. We also delineated the mechanism of action of the compound. 

Some reports on the anti-influenza properties of BBR are conflicting. For MDCK cells, Cecil et al. showed that BBR did not affect virus replication, while Wu et al. reported substantial inhibition in this culture model [19,20]. In our study, we observed noticeable influenza A virus inhibitory properties at high concentrations, similar to those obtained by Wu et al. [20]. The main difference between the studies was the concentration range used, as the effective concentration in the MDCK cells is unusually high. 

The results obtained with a second commonly used model based on A549 cells are also slightly confusing. While the activity of BBR has been reported in these cells [21], other reports show a strong cytotoxic activity of BBR towards cancerous cells, including A549 cells [10,30]. Indeed, while the effective concentration was rather low, the cytotoxic concentration was also low, and it was impossible to determine whether the observed effect is not an artifact. 

To sort out whether the antiviral effect of BBR has the potential to be used for treatment, we tested two further models employing non-cancerous cells originating from respiratory cells. First, mouse immortalized lung epithelial type 1 (LET1) line was used. Second, fully differentiated 3D primary human airway epithelium (HAE) cultures were employed. In both cases, we observed significant anti-influenza properties of BBR at non-toxic concentrations. These results confirm the anti-influenza properties of BBR, the low toxicity of the compound for the primary cells, and show the potency of the compound to inhibit the infection in the tissue.

In the subsequent step, we aimed to understand the molecular mechanism of action of the BBR on the influenza A virus. Functional assays demonstrated the effect of BBR on virus replication, and not the receptor binding or virus entry. The cell-type-dependent differences in the compound antiviral activity suggested that the compound likely acts on the host cell, and not the virus itself. For this reason, we made an effort to link the known activities of BBR with pathways that are important for the replication of the influenza A virus. Two recently published reports show BBR-mediated inhibition of the Chikungunya virus (CHIKV) and the enterovirus 71, which was caused by interference with the virus-induced mitogen-activated protein kinase (MAPK) signaling pathways, including extracellular signal-related kinase (ERK) [35,36]. It is also known that the active form of ERK1 (p-ERK1) is required for the nuclear export of the influenza A virus RNP complex during the infection [37] and that this pathway is modulated during the infection. First, the ERK pathway is upregulated shortly after the infection, to rapidly decrease after the first hour [38]. Next, the pathway is upregulated again at the late stage of the infection, when RNP must be exported from the nucleus to assemble into progeny virions [37]. ERK inhibition with a specific inhibitor U0126 impairs RNP transport and results in retention of RNPs inside the nucleus, and consequent inhibition of viral replication [37]. We verified if BBR exhibited activity similar to that of U0126. Confocal microscopy revealed a similar RNP retention within the cell nuclei in both BBR-treated and MAPK/ERK inhibitor U0126-treated A549 cells (Figure 5). Accumulation of the nucleoprotein was also observed when nuclear fractions of cells treated with BBR and U0126 were analyzed (Figure 6). The literature does not link the specific MAPK/ERK inhibition and the activity of BBR in A549 cells [39,40], which suggests that the antiviral effect is specific. 

In our studies, we used BBR diluted in DMSO. While this solvent is convenient for use in cell culture, regulatory agencies recommend it only for topical use [41,42]. Considering the route of administration of anti-influenza drugs (oral, inhalation, or intravenous) [43], other solvents should be considered, which may include conjugates or nanocages. Such new formulations may also increase the bioavailability of BBR [44].

Summarizing, our results demonstrate inhibition of the influenza A virus by BBR at non-toxic concentrations. The antiviral effect differs between cell lines, but we confirmed its relevance using the most advanced model of the human respiratory epithelium. A subsequent study revealed that the inhibition of the influenza A virus replication results from BBR-specific inhibition of the MAPK/ERK1 pathway, required for the effective production of progeny virions. It is worth noting that due to its mechanism of action, the emergence of resistant mutants is less likely than for commonly used inhibitors targeting viral proteins. 

## Figures and Tables

**Table 1:** Inhibition of influenza A virus in different cellular models. Experimental values of half-maximal inhibitory concentration (IC_50_), half-maximal toxic concentration (TC_50_) and selectivity index (SI) of berberine (BBR) versus influenza A virus replication in different cell lines. A549: adenocarcinoma human alveolar basal epithelial cells; MDCK: Madin–Darby canine kidney cells; LET1: lung epithelial type I cell line; HAE: human airway epithelial cells. For HAE cultures no TC_50_ and SI values were measured (indicated as “-“).

	IC_50_	TC_50_	SI
**A549**	17 µM (0.006 µg/mL)	107 µM (0.036 µg/mL)	6
**MDCK**	52 µM (0.017 µg/mL)	1035 µM (0.350 µg/mL)	20
**LET1**	4 µM (0.001 µg/mL)	521 µM (0.176 µg/mL)	123
**HAE**	16 µM (0.005 µg/mL)	-	-

## References

[1] Shaw M.L., Palese P. (2007). Orthomyxoviridae: The viruses and their replication. Fields Virol..

[2] Morens D.M., Taubenberger J.K., Harvey H.A., Memoli M.J. (2010). The 1918 influenza pandemic: Lessons for 2009 and the future. Crit. Care Med..

[3] Dawood F.S., Iuliano A.D., Reed C., Meltzer M.I., Shay D.K., Cheng P.Y., Bandaranayake D., Breiman R.F., Brooks W.A., Buchy P. (2012). Estimated global mortality associated with the first 12 months of 2009 pandemic influenza A H1N1 virus circulation: A modelling study. Lancet Infect. Dis..

[4] Nair H., Brooks W.A., Katz M., Roca A., Berkley J.A., Madhi S.A., Simmerman J.M., Gordon A., Sato M., Howie S. (2011). Global burden of respiratory infections due to seasonal influenza in young children: A systematic review and meta-analysis. Lancet.

[5] Reperant L.A., Moesker F.M., Osterhaus A.D.M.E. (2016). Influenza: From zoonosis to pandemic. ERJ Open Res..

[6] Thompson M.G., Pierse N., Sue Huang Q., Prasad N., Duque J., Claire Newbern E., Baker M.G., Turner N., McArthur C. (2018). Influenza vaccine effectiveness in preventing influenza-associated intensive care admissions and attenuating severe disease among adults in New Zealand 2012–2015. Vaccine.

[7] Ferdinands J.M., Olsho L.E.W., Agan A.A., Bhat N., Sullivan R.M., Hall M., Mourani P.M., Thompson M., Randolph A.G. (2014). Effectiveness of influenza vaccine against life-threatening RT-PCR-confirmed influenza illness in US children, 2010-2012. J. Infect. Dis..

[8] Hussain M., Galvin H.D., Haw T.Y., Nutsford A.N., Husain M. (2017). Drug resistance in influenza a virus: The epidemiology and management. Infect. Drug Resist..

[9] Hayden F.G., Sugaya N., Hirotsu N., Lee N., de Jong M.D., Hurt A.C., Ishida T., Sekino H., Yamada K., Portsmouth S. (2018). Baloxavir Marboxil for Uncomplicated Influenza in Adults and Adolescents. N. Engl. J. Med..

[10] Gan R. (2012). Bioactivities of Berberine: An Update. Int. J. Mod. Biol. Med..

[11] Neag M.A., Mocan A., Echeverría J., Pop R.M., Bocsan C.I., Crisan G., Buzoianu A.D. (2018). Berberine: Botanical Occurrence, traditional uses, extraction methods, and relevance in cardiovascular, metabolic, hepatic, and renal disorders. Front. Pharmacol..

[12] Imenshahidi M., Hosseinzadeh H. (2016). Berberis Vulgaris and Berberine: An Update Review. Phyther. Res..

[13] Kumar A., Ekavali, Chopra K., Mukherjee M., Pottabathini R., Dhull D.K. (2015). Current knowledge and pharmacological profile of berberine: An update. Eur. J. Pharmacol..

[14] Qiu S., Sun H., Zhang A.H., Xu H.Y., Yan G.L., Han Y., Wang X.J. (2014). Natural alkaloids: Basic aspects, biological roles, and future perspectives. Chin. J. Nat. Med..

[15] Batista M.N., Braga A.C.S., Fernandes Campos G.R., Michel Souza M., de Matos R.P.A., Zara Lopes T., Maria Candido N., Duarte Lima M.L., Cristina Machado F., de Andrade S.T.Q. (2019). Natural products isolated from oriental medicinal herbs inactivate zika virus. Viruses.

[16] Robinson C.L., Chong A.C.N., Ashbrook A.W., Jeng G., Jin J., Chen H., Tang E.I., Martin L.A., Kim R.S., Kenyon R.M. (2018). Male germ cells support long-term propagation of Zika virus. Nat. Commun..

[17] Marchant D., Singhera G.K., Utokaparch S., Hackett T.L., Boyd J.H., Luo Z., Si X., Dorscheid D.R., McManus B.M., Hegele R.G. (2010). Toll-Like Receptor 4-Mediated Activation of p38 Mitogen-Activated Protein Kinase Is a Determinant of Respiratory Virus Entry and Tropism. J. Virol..

[18] Zou K., Li Z., Zhang Y., Zhang H.Y., Li B., Zhu W.L., Shi J.Y., Jia Q., Li Y.M. (2017). Advances in the study of berberine and its derivatives: A focus on anti-inflammatory and anti-tumor effects in the digestive system. Acta Pharmacol. Sin..

[19] Cecil C.E., Davis J.M., Cech N.B., Laster S.M. (2011). Inhibition of H1N1 influenza A virus growth and induction of inflammatory mediators by the isoquinoline alkaloid berberine and extracts of goldenseal (Hydrastis canadensis). Int. Immunopharmacol..

[20] Wu Y., Li J., Kim Y., Wu J., Wang Q., Hao Y. (2011). In vivo and in vitro antiviral effects of berberine on influenza virus. Chin. J. Integr. Med..

[21] Yan Y.Q., Fu Y.J., Wu S., Qin H.Q., Zhen X., Song B.M., Weng Y.S., Wang P.C., Chen X.Y., Jiang Z.Y. (2018). Anti-influenza activity of berberine improves prognosis by reducing viral replication in mice. Phyther. Res..

[22] Ciejka J., Milewska A., Wytrwal M., Wojarski J., Golda A., Ochman M., Nowakowska M., Szczubialka K., Pyrc K. (2016). Novel polyanions inhibiting replication of influenza viruses. Antimicrob. Agents Chemother..

[23] Ciejka J., Botwina P., Nowakowska M., Szczubiałka K., Pyrc K. (2019). Synthetic sulfonated derivatives of poly (allylamine hydrochloride) as inhibitors of human metapneumovirus. PLoS ONE.

[24] Reed L.J., Muench H. (1938). A simple method of estimating fifty per cent endpoints. Am. J. Hyg..

[25] Harden E.A., Falshaw R., Carnachan S.M., Kern E.R., Prichard M.N. (2009). Virucidal activity of polysaccharide extracts from four algal species against herpes simplex virus. Antiviral Res..

[26] Schindelin J., Arganda-Carreras I., Frise E., Kaynig V., Longair M., Pietzsch T., Preibisch S., Rueden C., Saalfeld S., Schmid B. (2012). Fiji: An open-source platform for biological-image analysis. Nat. Methods.

[27] Kula A., Guerra J., Knezevich A., Kleva D., Myers M.P., Marcello A. (2011). Characterization of the HIV-1 RNA associated proteome identifies Matrin 3 as a nuclear cofactor of Rev function. Retrovirology.

[28] Kim J.H., Weeratunga P., Kim M.S., Nikapitiya C., Lee B.H., Uddin M.B., Kim T.H., Yoon J.E., Park C., Ma J.Y. (2016). Inhibitory effects of an aqueous extract from Cortex Phellodendri on the growth and replication of broad-spectrum of viruses in vitro and in vivo. BMC Complement. Altern. Med..

[29] Qi H.W., Xin L.Y., Xu X., Ji X.X., Fan L.H. (2014). Epithelial-to-mesenchymal transition markers to predict response of Berberine in suppressing lung cancer invasion and metastasis. J. Transl. Med..

[30] Li J., Liu F., Jiang S., Liu J., Chen X., Zhang S., Zhao H. (2018). Berberine hydrochloride inhibits cell proliferation and promotes apoptosis of non-small cell lung cancer via the suppression of the MMP2 and Bcl-2/bax signaling pathways. Oncol. Lett..

[31] Rosenberger C.M., Podyminogin R.L., Askovich P.S., Navarro G., Kaiser S.M., Sanders C.J., McClaren J.L., Tam V.C., Dash P., Noonan J.G. (2014). Characterization of innate responses to influenza virus infection in a novel lung type I epithelial cell model. J. Gen. Virol..

[32] Milewska A., Nowak P., Stozek K., Potempa J., Pyrc K., Zarebski M. (2014). Human coronavirus NL63 utilizes heparan sulfate proteoglycans for attachment to target cells. J. Virol..

[33] Milewska A., Nowak P., Owczarek K., Szczepanski A., Zarebski M., Hoang A., Berniak K., Wojarski J., Zeglen S., Baster Z. (2017). Entry of Human Coronavirus NL63 into the Cell. J. Virol..

[34] Gaur P., Munjhal A., Lal S.K. (2011). Influenza virus and cell signaling pathways. Med. Sci. Monit..

[35] Varghese F.S., Ahola T., Thaa B., McInerney G.M., Amrun S.N., Simarmata D., Ng L.F.P., Rausalu K., Merits A., Nyman T.A. (2016). The antiviral alkaloid berberine reduces Chikungunya virus-induced mitogen-activated protein kinase signaling. J. Virol..

[36] Wang H., Li K., Ma L., Wu S., Hu J., Yan H., Jiang J., Li Y. (2017). Berberine inhibits enterovirus 71 replication by downregulating the MEK/ERK signaling pathway and autophagy. Virol. J..

[37] Pleschka S., Wolff T., Ehrhardt C., Hobom G., Planz O., Rapp U.R., Ludwig S. (2001). Influenza virus propagation is impaired by inhibition of the Raf/MEK/ERK signalling cascade. Nat. Cell Biol..

[38] Ehrhardt C., Marjuki H., Wolff T., Nürnberg B., Planz O., Pleschka S., Ludwig S. (2006). Bivalent role of the phosphatidylinositol-3-kinase (PI3K) during influenza virus infection and host cell defence. Cell. Microbiol..

[39] Warmka J.K., Solberg E.L., Zeliadt N.A., Srinivasan B., Charlson A.T., Xing C., Wattenberg E.V. (2012). Inhibition of mitogen activated protein kinases increases the sensitivity of A549 lung cancer cells to the cytotoxicity induced by a kava chalcone analog. Biochem. Biophys. Res. Commun..

[40] Lu C.L., Lin H.I., Chen B.F., Jow G.M. (2016). Beauvericin-induced cell apoptosis through the mitogen-activated protein kinase pathway in human nonsmall cell lung cancer A549 cells. J. Toxicol. Sci..

[41] Verheijen M., Lienhard M., Schrooders Y., Clayton O., Nudischer R., Boerno S., Timmermann B., Selevsek N., Schlapbach R., Gmuender H. (2019). DMSO induces drastic changes in human cellular processes and epigenetic landscape in vitro. Sci. Rep..

[42] Smith E.R., Hadidian Z., Mason M.M. (1967). THE SINGLE–AND REPEATED–DOSE TOXICITY OF DIMETHYL SULFOXIDE. Ann. N. Y. Acad. Sci..

[43] Uyeki T.M., Bernstein H.H., Bradley J.S., Englund J.A., File T.M., Fry A.M., Gravenstein S., Hayden F.G., Harper S.A., Hirshon J.M. (2019). Clinical Practice Guidelines by the Infectious Diseases Society of America: 2018 Update on Diagnosis, Treatment, Chemoprophylaxis, and Institutional Outbreak Management of Seasonal Influenzaa. Clin. Infect. Dis..

[44] Godugu C., Patel A.R., Doddapaneni R., Somagoni J., Singh M. (2014). Approaches to improve the oral bioavailability and effects of novel anticancer drugs berberine and betulinic acid. PLoS ONE.

